# The association between peritraumatic distress, perceived stress, depression in pregnancy, and NR3C1 DNA methylation among Chinese pregnant women who experienced COVID-19 lockdown

**DOI:** 10.3389/fimmu.2022.966522

**Published:** 2022-08-25

**Authors:** Liqing Wei, Xiaohong Ying, Mengxi Zhai, Jiayu Li, Dan Liu, Xin Liu, Bin Yu, Hong Yan

**Affiliations:** ^1^ Department of Epidemiology and Health Statistics, School of Public Health, Wuhan University, Wuhan, China; ^2^ Population and Health Research Center, Wuhan University, Wuhan, China

**Keywords:** pregnant women, perceived stress, peritraumatic distress, depression, DNA methylation, glucocorticoid receptor, NR3C1

## Abstract

Prenatal stress can affect pregnant women in an epigenetic way during the critical period of conception of their offspring. The study aims to investigate the relationship between peritraumatic distress, prenatal perceived stress, depression, and glucocorticoid receptor (NR3C1) DNA methylation among pregnant women who experienced COVID-19 lockdown in China. Study data were collected from 30 pregnant women in Wuhan and Huanggang, China. The Peritraumatic Distress Inventory was used to measure peritraumatic distress, the Edinburgh Postnatal Depression Scale was used to measure depressive symptoms, and the Perceived Stress Scale was used to measure perceived stress. DNA methylation in the exon 1F promoter region of NR3C1 gene from the venous blood mononuclear cell genome was characterized by bisulfite sequencing. Correlation and linear regression were used for data analysis. The mean level of peritraumatic distress, perceived stress, and depression was 6.30 (SD = 5.09), 6.50 (SD = 5.41), and 6.60 (SD = 4.85), respectively, with 23.33% of pregnant women being depressed. The mean NR3C1 methylation was 0.65 (SD = 0.22). Prenatal depression was positively correlated with the degree of methylation in venous blood from the mother (r = 0.59, p = 0.001), and depression predicted methylation of NR3C1 gene at the CpG 8 site (β = 0.05, p = 0.03). No association was found between peritraumatic distress as well as perceived stress and methylation of NR3C1. NR3C1 gene was susceptible to epigenetic modification of DNA methylation in the context of prenatal stress, and maternal depression was associated with increased NR3C1 methylation among women who experienced COVID-19 lockdown.

## Introduction

In December 2019, the outbreak of coronavirus disease-2019 (COVID-19) caused by severe acute respiratory syndrome coronavirus 2 (SARS-CoV-2) occurred in Wuhan, China (1–3). To slow down and finally end the spread of COVID-19, the Chinese government adopted a measure of lockdown in areas with outbreaks. Previous studies suggested that stress caused by negative events, such as COVID-19 lockdown, can affect vulnerable people, particularly pregnant women (4, 5). Data from several countries showed higher levels of maternal stress and more pronounced increases in depression, anxiety, and negative emotions during the COVID-19 pandemic (6–8). A multicenter study conducted in China also showed similar results (9).

Poor mental health status (e.g., prenatal stress and depression) during pregnancy has been reported to be associated with many negative health outcomes, including poor nutrition (10), suicidality (11) for mothers, preterm delivery (12), and impaired cognitive development (13) for offspring. One study conducted in Peru indicated that depressed pregnant women had a higher risk of suicidal ideation than non-depressed pregnant women (14). Previous research has shown that certain obstetric complications (15) and lower exclusive breastfeeding rates (16) were directly related to prenatal maternal stress, such as perceived stress and depression. Obstetric complications due to prenatal stress and depression include miscarriage (17), preeclampsia (18), congenital malformations (19), preterm birth (18), and low birth weight of the fetus (20). Thus, it is essential to pay more attention to the mental health status of pregnant women.

However, the underlying mechanisms by which prenatal poor mental health causes these negative health outcomes are still being debated. Disturbances in the activity of the hypothalamic–pituitary–adrenal (HPA) axis may be used as a theoretical basis for understanding the adverse outcomes. During pregnancy, women are exposed to various stressors that may be related to HPA activation. In studies of major depressive disorder, an increase in HPA axis activity was found in depressed patients with elevated basal cortisol (21, 22). The affinity of glucocorticoid receptors (GRs) for cortisol is relatively low and therefore is primarily responsive to continuous and greater levels of cortisol (23). The HPA axis can be activated throughout a lifetime by psychosocial stress (7). Sustained stressful conditions have chronic and ultimately damaging effects on the body’s stress regulation through the downregulation of GR by epigenetic mechanisms (24).

Recent studies have shown that the link between maternal prenatal mental health status and HPA axis function may be mediated by DNA methylation, a possible epigenetic mechanism that eventually leads to adverse outcomes for the mother (25) and infant (26). DNA methylation regulates gene expression but does not change the sequence of DNA. The methyl group is transferred to the C5 position of cytosine, forming 5-methylcytosine, which is the process of DNA methylation. DNA methylation within the C-phosphate-G (CpG) dinucleotide context is a pivotal factor in regulating gene expression. Previous studies have found that several DNA methylation changes may be related to the function of the fetal HPA axis (27), including the nuclear receptor subfamily 3, group C, member 1 (NR3C1) DNA methylation (28). GR production is encoded through NR3C1, which contributes to the inhibition of cortisol production. NR3C1 DNA methylation homeostasis maintains the normal function of the HPA axis negative feedback loop and protects the fetus from excessive cortisol exposure (29). Empirical studies showed that prenatal stress led to methylation of NR3C1 gene in both mother and fetus and that elevated methylation was associated with poor fetal outcomes and risk of disease in offspring’s adulthood (30, 31). Another study in the United States also found that maternal depression during pregnancy was associated with increased placental NR3C1 CpG 2 methylation and higher levels of infant lethargy, lower muscle tone, and poorer self-regulation (32).

Previous studies on the effects of stress on maternal DNA methylation mainly focused on the stress of war in war-torn African countries (30, 33) or the stress of discrimination among Latina mothers in the United States (34, 35). COVID-19 presents a new challenge to humanity as a completely new disease that causes stress unlike the above types of stress. With regard to the first COVID-19 wave pandemic, which resulted in a large number of infected individuals and deaths, considerable uncertainty remains about the characteristics of the virus and the infection. Against this background, the Chinese government took measures to lock down the city to stop the spread of SARS-CoV-2. During the lockdown period, the residents confined their activities to their households. The lack of an effective vaccine and treatment during the first COVID-19 wave also increased the public’s fear of infection. A study has shown that prenatal stress related to COVID-19 is associated with methylation of the gene in infants (36) and that it is possible that the COVID-19-related stress exposure during this special period may also exert effects on the NR3C1 DNA methylation in pregnant women.

In the present study, we aimed to investigate whether and to what extent prenatal peritraumatic distress, perceived stress, and depression in Chinese pregnant women during COVID-19 lockdown affect maternal NR3C1 DNA methylation. The findings of the study will provide valuable evidence for future effective intervention and prevention programs targeting poor mental health among pregnant women in the context of the COVID-19 pandemic or other public health emergencies.

## Materials and methods

### Participants and sampling

The study included 30 randomly selected participants from the Maternal and Child Health Cohort Study of 293 pregnant women (37). Participants in the study were pregnant women who were 18–49 years old, at their second and third trimesters of pregnancy, without a diagnosis of mental disorders and medical complications prior to and during pregnancy. All mothers who participated in this study tested negative for COVID-19. These participants were recruited from Huganggang Maternal and Child Health Hospital and Wuhan Zhenai Maternal Hospital from May to July 2020. Between late January and early April 2020, residents of both Wuhan and Huanggang experienced a lockdown period of approximately 2 months. Wuhan is the capital city of Hubei Province, with a population of 11.21 million and a per capita GDP of 145545 RMB. Huanggang City is adjacent to Wuhan with a population of 6.33 million per capita GDP of 36,685 RMB. Only participants who agreed to participate and signed the informed consent were enrolled, and the participation was voluntary and confidential. Pregnant women have the right to withdraw or quit at any time. The differences in key outcome variables between the two cities were compared, and no significant differences were found.

### Data collection

The study data consisted of two parts, including a survey (maternal mental health survey) and blood samples from mothers. The survey was delivered through a paper–pencil approach. Participants were asked to complete the survey independently in a private room. The trained data collectors were prepared to provide help if needed. Upon completion, a data collector would double-check the questionnaire and make sure every part was completed.

Regarding the blood samples, venous blood samples from mothers were collected on the day of childbirth. Venous blood pretreated with EDTA was transferred to lyophilized tubes and then placed in a −80°C refrigerator. Upon completing the survey and collecting blood samples, participants would receive a reward of 30 RMB. The study was approved by Wuhan University Medical Ethics Committee.

### Measures

#### Peritraumatic distress inventory

The maternal peritraumatic distress related to the pandemic was measured using the Peritraumatic Distress Inventory (PDI) (38). The scale consists of 13 items, and typical items include “I felt sadness and grief” and “I thought I might die”. Each item is measured using a 5-point Likert scale ranging from “0 = not at all true” to “4 = extremely true”. The total score of PDI was calculated with a higher score indicating severer peritraumatic distress in pregnant women. Cronbach’s alpha of PDI was 0.84 in the study.

#### Perceived stress

The maternal perceived stress was measured using the Perceived Stress Scale (PSS) (39). The scale consists of 10 items, and typical items include “In the last month, how often have you felt that you were unable to control the important things in your life?” and “In the last month, how often have you been able to control irritations in your life?”. Each item is measured using a 5-point Likert scale ranging from “0 = never” to “4 = very often”. The total score of PSS was calculated with a higher score indicating greater stress perceived by pregnant women. Cronbach’s alpha of PSS was 0.93 in the study.

#### Depression

Maternal depression was measured using the Edinburgh Postnatal Depression Scale (EPDS) (40). The scale consists of 10 items, and typical items include “I have been able to laugh and see the funny side of things” and “I have blamed myself unnecessarily when things went wrong”. Each item is measured using a 4-point Likert scale ranging from “0 = not at all” to “3 = very often”. The total score of EPDS was calculated with a higher score indicating a greater level of depressive symptoms. The EPDS score was categorized into depression and non-depression with a cutoff value of 10. Cronbach’s alpha of EPDS was 0.65 in the study.

#### Demographic variables

The demographic variables in the study included age (in years and categorized into ≤25, 26–30, and >30), city (Wuhan/Huanggang), residence (rural/urban), education (middle school or lower, high school, and college or higher), husband’s education (middle school or lower, high school, and college or higher), employment status (full-time job and part-time job/others), monthly income (in RMB, ≤2,000, 2,001–5,000, and >5,000), annual household income (in RMB, ≤10,000, 10,001–20,000, and >20,000), and family size (in the number of persons, ≤3, 4, and ≥5).

#### DNA extraction and bisulfite modification

Venous blood samples collected from 30 mothers were used to assess the methylation of the GR-1F promoter of the NR3C1 exon. Genomic DNA was extracted using the TIANamp Blood DNA Midi Kit (Tiangen, China). Quantification of purified DNA was carried out using an ND-1000 spectrophotometer (NanoDrop, Wilmington, DE, USA). Samples of DNA (500 ng) were bisulfated using the QIAGEN DNA methylation kit (QIAGEN, Valencia, CA, USA) and stored at −80°C.

#### Cytosine methylation assessment

**Figure 1:**
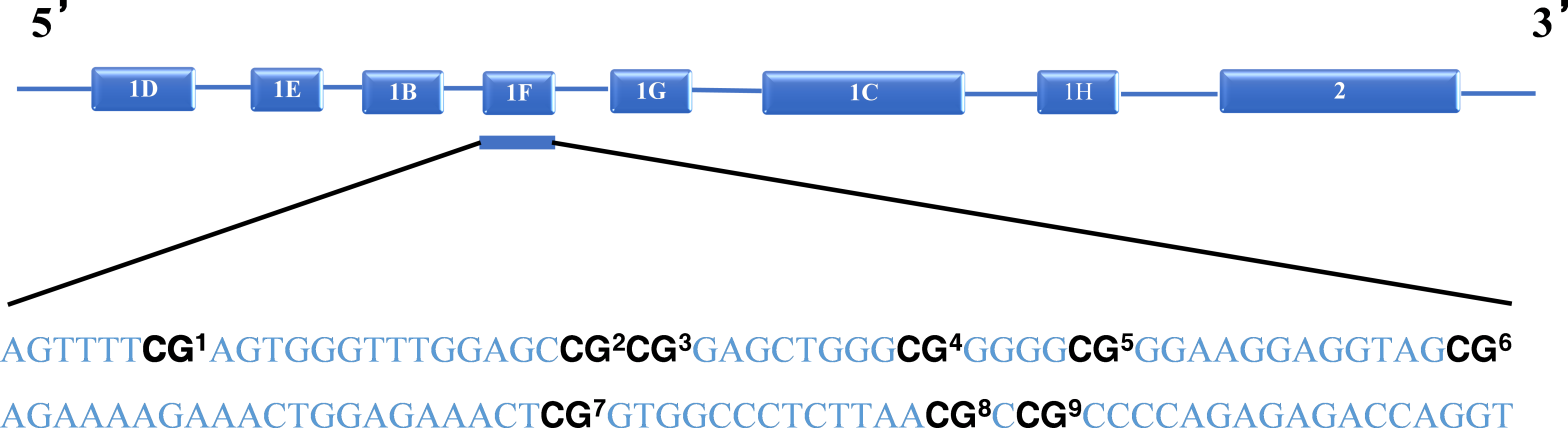
Schematic representation of NR3C1 gene and the CpG sites analyzed.

Nine C-phosphate-G (CpG) sites of the GR-1F promoter of NR3C1 were used as the target CpG site of methylation (**Figure 1**). The number of methylated clones per CpG site was converted to percentages, and the methylation percentages of the nine CpG sites were summed as a total methylation percentage. The primers for amplification were forward: 5′-GTTAAGGGGTAGAGCGAGT TTT-3′, and reverse: 5′-AAAACCGACCTAATCTCTCTAAAAC-3′. A bisulfite conversion control was used for each sequencing read for NR3C1. In order to call the methylation level of the samples, for all samples analyzed, the bisulfite conversion rate was >95%. A triplicate of all assays was carried out on the identical DNA template converted by bisulfite. In case any of the replicates varied by >10%, the assay was repeated for that sample.

### Statistical analysis

Descriptive analyses (e.g., frequency, percentage, mean, and standard deviation) were used to describe the sample characteristics. Student’s t-test and ANOVA were used to compare the methylation levels across demographic variables and different levels of depression. Correlation analysis was used to associate the peritraumatic distress, perceived stress, depression, and NR3C1 gene methylation. Mixed-effects modeling analysis was used to investigate the association between peritraumatic distress, perceived stress, depression, and methylation in different CpG sites when controlling for covariates and including sampling location (Wuhan and Huanggang) as a random effect. The false discovery rate (FDR) method was performed to adjust the multiple testing. All statistical analyses were performed using the IBM SPSS version 25.0 (IBM Corp, New York, USA).

## Results

### Characteristics of the study sample

Results in **Table 1** show that the mean age of the study sample was 29.13 years (SD = 5.63). In total, 76.67% of participants came from Huanggang City. More than half of the participants (53.33%) came from rural areas, 40.00% had a college education or higher, 53.33% of the pregnant women’s husbands had a college education or higher, and 50% had a full-time job. Approximately 80% of participants earned less than 5,000 RMB (equivalent to $770) per month, only 26.67% had an annual household income >20,000 RMB (equivalent to $3,090), and 26.67% had a family size of ≤3 and 46.67% with ≥5. The mean peritraumatic distress of mothers was 6.30 (SD = 5.09). The mean perceived stress was 6.50 (SD = 5.41). The mean depression of mothers was 6.60 (SD = 4.85), and a total of 23.33% were depressed.

**Table 1:** Characteristics of the study sample.

Variables	Category	N	%
**Total**		30	100.00
**Age**			
	Mean (SD)	29.13	5.63
	21–25	9	30.00
	26–30	8	26.67
	>30	13	43.33
**City**			
	Huanggang	23	76.67
	Wuhan	7	23.33
**Residence**			
	Urban	14	46.67
	Rural	16	53.33
**Education**			
	Middle school or lower	10	33.33
	High school	8	26.67
	College or higher	12	40.00
**Husband’s education**			
	Middle school or lower	10	33.33
	High school	4	13.33
	College or higher	16	53.33
**Employment status**			
	Full-time job	15	50.00
	Part-time job/others	15	50.00
**Monthly income, RMB**			
	≤2,000	10	33.33
	2,001–5,000	14	46.67
	>5,000	6	20.00
**Annual household income, RMB**			
	≤10,000	13	43.33
	10,001–20,000	9	30.00
	>20,000	8	26.67
**Family size**			
	≤3	8	26.67
	4	8	26.67
	≥5	14	46.67
**Peritraumatic distress**	Mean (SD)	6.30	5.09
**Perceived stress**	Mean (SD)	6.50	5.41
**Depression**			
	Yes	7	23.33
	No	23	76.67
**Depressive symptoms**	Mean (SD)	6.60	4.85

### Differences in NR3C1 DNA methylation across demographic groups

Results in **Table 2** show that among the total sample, the mean NR3C1 gene methylation was 0.65 (SD = 0.22). There were no significant differences in NR3C1 DNA methylation across demographic groups.

**Table 2:** Levels of NR3C1 methylation among pregnant women, overall and by demographic groups, and levels of depression.

Variables	Methylation (%)	F/t	p
**Total**	64.48 (21.58)		
**Age**		0.46	0.64
21–25	67.90 (18.79)		
26–30	58.33 (25.02)		
>30	65.81 (21.97)		
**Residence**		−0.03	0.98
Urban	64.29 (20.06)		
Rural	64.58 (23.38)		
**Education**		0.30	0.74
Junior high school or lower	67.78 (21.88)		
High school or junior college	59.72 (25.15)		
College or higher	64.81 (20.01)		
**Husband education**		0.10	0.90
Junior high school or lower	66.67 (22.22)		
High school or junior college	61.11 (26.45)		
College or higher	63.89 (21.28)		
**Employment status**		3.07	0.09
Full-time job	71.11 (16.69)		
Part-time job/others	57.78 (24.20)		
**Monthly income**		2.92	0.07
≤2,000	57.78 (23.31)		
2,001–5,000	73.81 (14.85)		
>5,000	53.70 (25.74)		
**Annual family income**		0.04	0.96
≤10,000	65.81 (22.43)		
10,001–20,000	62.96 (19.25)		
>20,000	63.89 (25.02)		
**Family size**		0.71	0.50
≤3	65.28 (20.09)		
4	56.94 (28.75)		
≥5	68.25 (17.89)		
**Depression**		−2.13	0.06
Yes	77.78 (18.14)		
No	60.39 (21.14)		

### Associations between NR3C1 DNA methylation and peritraumatic distress, perceived stress, and depression

Results in the lower panel of **Table 2** show that pregnant women who were depressed had a higher NR3C1 DNA methylation level than women who were not depressed with a marginal significance level (t = −2.13, p = 0.06).

**Figure 2:**
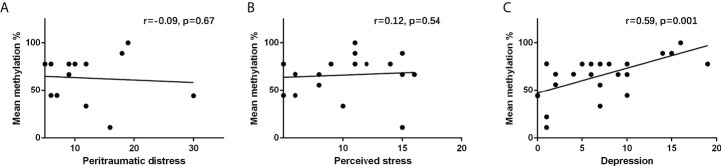
Correlation between **(A)** peritraumatic distress and NR3C1 gene methylation, **(B)** perceived stress and NR3C1 gene methylation, and **(C)** depression and NR3C1 gene methylation.

Results in **Figures 2A, B** show that NR3C1 methylation was not significantly correlated with peritraumatic distress (r = −0.09, p = 0.67) and perceived stress (r = 0.12, p = 0.54). Results in **Figure 2C** show that NR3C1 methylation was significantly correlated with depression (r = 0.59, p = 0.001).

Results in **Table 3** show that none of them were associated with peritraumatic distress or perceived stress among nine CpG sites in NR3C1 DNA promotor. Still, one of them (CpG 8) was found to be significantly associated with depression (β = 0.05 p = 0.03, R^2^ = 0.21).

**Table 3:** Effects of peritraumatic distress, perceived stress and depression on NR3C1 gene CpG sites methylation among pregnant women.

NR3C1 gene CpG site	Beta	SE	p	p^#^	Adjusted R^2^
**Peritraumatic distress**					
CpG 1	−0.01	0.01	0.36	0.63	−0.01
CpG 2	−0.02	0.01	0.09	0.54	0.07
CpG 3	−0.02	0.01	0.12	0.54	0.05
CpG 4	−0.01	0.01	0.42	0.63	−0.01
CpG 5	0.001	0.01	0.91	0.94	−0.04
CpG 6	0.001	0.01	0.94	0.94	−0.04
CpG 7	0.02	0.01	0.18	0.54	0.03
CpG 8	0.01	0.01	0.73	0.94	−0.03
CpG 9	0.01	0.01	0.35	0.63	−0.003
**Perceived stress**					
CpG 1	0.02	0.02	0.28	0.92	0.01
CpG 2	0.003	0.01	0.78	0.92	−0.03
CpG 3	−0.01	0.01	0.67	0.92	−0.03
CpG 4	0.002	0.01	0.89	0.92	−0.03
CpG 5	0.01	0.01	0.41	0.92	−0.01
CpG 6	0.01	0.01	0.72	0.92	−0.03
CpG 7	0.01	0.02	0.36	0.92	−0.01
CpG 8	0.03	0.01	0.06	0.50	0.09
CpG 9	−0.002	0.02	0.92	0.92	−0.04
**Depression**					
CpG 1	0.02	0.02	0.22	0.25	0.02
CpG 2	0.03	0.02	0.07	0.16	0.08
CpG 3	0.03	0.02	0.13	0.19	0.05
CpG 4	0.02	0.02	0.17	0.22	0.04
CpG 5	0.02	0.01	0.11	0.19	0.03
CpG 6	0.03	0.01	0.03	0.12	0.13
CpG 7	−0.003	0.02	0.87	0.87	−0.03
CpG 8	**0.05**	**0.02**	**0.003**	**0.03**	**0.21**
CpG 9	0.04	0.02	0.04	0.12	0.13

The regression model was controlled for age, city, and residence.

**^#^** Multiple testing was adjusted using the false discovery rate (FDR) method.

Bold values indicate that CpG8 was found to be significantly associated with depression.

## Discussion

To the best of our knowledge, this is the first study to investigate the relationship between prenatal stress, depression, and methylation of NR3C1 gene in venous blood of pregnant Chinese women who experienced COVID-19 lockdown. The results of this study add new data to deepen our understanding of the role of maternal mental health in maternal gene methylation changes during infectious disease epidemics.

Some studies reported that COVID-19 lockdown causes elevated stress and depression in pregnant women (5–8), but we did not find elevated levels of maternal stress and depression as compared to foreign studies (7, 41–43) as well as studies conducted in China before the COVID-19 pandemic (44, 45). These findings suggest that the citywide lockdown did not significantly increase prenatal stress and depression. A possible explanation may include that Hubei Province and Wuhan City received great social, emotional, and instrumental support nationwide, improving their capacity against the COVID-19-related stress (46, 47). In addition, in the context of traditional Chinese values, pregnant women in China receive more care and social support from their families and society, thus, they can better cope with stress (48). The study’s findings indicated that prenatal peritraumatic distress or perceived stress was not significantly associated with maternal NR3C1 gene methylation. This is inconsistent with a previous study conducted in the Democratic Republic of Congo that showed a significant association between mean methylation and peritraumatic distress or mundane stress (30). Furthermore, a study conducted in the United States showed a significantly strong association between everyday discrimination and NR3C1 gene methylation (35). Meanwhile, many studies conducted in Africa also found that prenatal war stress was associated with NR3C1 gene methylation (30, 33). It is likely that the difference may be attributable to the type and level of stress experienced by the women in this study. The pregnant Chinese women in this study experienced stress during the COVID-19 lockdown period, but not as much as the stress of war in Africa or the stress of discrimination experienced by Latina women in the United States. Although the effect of peritraumatic distress and perceived stress on DNA methylation was insignificant, as a prenatal stressor, maternal stress, if not being coped with appropriately, may subsequently lead to a range of adverse health effects on both the mothers and their fetus.

Compared with prenatal stress, maternal prenatal depression showed a different map. The findings showed that pregnant women who were depressed had significantly higher levels of NR3C1 gene methylation. There may be a dose–response effect, with higher levels of methylation in pregnant women with more severe depressive symptoms. However, a recent study conducted among 163 mother–infant dyads in the United States reported that methylation of NR3C1 gene was not associated with depressive symptoms (49). The sample used in the US study was cord blood, whereas venous blood from the mother was used in our study. The difference in tissues may have contributed to the inconsistent results. Further, our study demonstrated a correlation between prenatal depression exposure and DNA methylation at a specific CpG 8 site of NR3C1 gene. No associations between other CpG sites and methylation levels were found. The analytic sites for NR3C1 gene in the US study were in the CpG island shore region, whereas our analytic site was in the exon 1F promoter region of NR3C1 gene. The difference in detection sites may affect the results.

The relationship between NR3C1 methylation and depression has been validated in studies of mothers and their infants (29, 32), with children of mothers with depression having higher DNA methylation of NR3C1 gene than children of mothers without depression. While most studies have focused on the relationship between maternal prenatal mental health and methylation of NR3C1 gene in the offspring, the present study provided an alternative approach to investigate the relationship between prenatal depression and maternal NR3C1 methylation. The methylation changes in the offspring have resulted from persistent elevations in cortisol caused by dysfunction of the mother’s HPA axis, which may be attributable to the maternal gene methylation.

In the present study, we also examined whether sociodemographic factors were associated with DNA methylation of NR3C1, but we found that maternal NR3C1 DNA methylation was not associated with age, residence, education, husband’s education, employment status, monthly income, annual family income, or family size. The results may be attributed to the relatively homogenous sample in gene levels, although they were differed in sociodemographic factors.

There are several limitations to this study. Firstly, the study was conducted among 30 pregnant women, which is a limited sample size. Although many studies on methylation have relatively similar sample sizes (50–52), a larger sample size will be more convincing to interpret the results. Despite the limited sample size, the range of reported depressive symptom scores was broad. Some mothers scored very low on depression, suggesting that the sample was not exclusive to highly depressed mothers. Secondly, peritraumatic distress, perceived stress, and depression were measured using self-reported scales, so recall bias may exist. Finally, our study examined only nine CpG sites across CpG islands in the NR3C1 exon. The possibility that prenatal depression may have differential effects on different CpG sites could be optimized by using genome-wide bisulfite sequencing in future studies.

The results of this study showed for the first time that prenatal maternal depression was associated with the methylation of NR3C1 gene among pregnant women in China. Further studies in a longitudinal cohort are needed to understand the subsequent effects of prenatal mental health of pregnant women who experienced COVID-19 lockdown on maternal and fetal outcomes.

## Data availability statement

The raw data supporting the conclusions of this article will be made available by the authors, without undue reservation.

## Ethics statement

The studies involving human participants were reviewed and approved by Medical Ethics Committee at Wuhan University. The patients/participants provided their written informed consent to participate in this study.

## Author contributions

HY had full access to all of the data in the study and takes responsibility for the integrity of the data and the accuracy of the data analysis. Concept and design: LW, HY, and BY. Acquisition, analysis, or interpretation of data: XY, MZ, JL, DL, and XL. Drafting of the manuscript: LW and BY. Critical revision of the manuscript for important intellectual content: HY and BY. Statistical analysis: LW. Administrative, technical, or material support: LW and XY. All authors contributed to the article and approved the submitted version.

## Acknowledgments

We thank all participants in this study for sharing their life experiences and perceptions.

## Conflict of interest

The authors declare that the research was conducted in the absence of any commercial or financial relationships that could be construed as a potential conflict of interest.

## Publisher’s note

All claims expressed in this article are solely those of the authors and do not necessarily represent those of their affiliated organizations, or those of the publisher, the editors and the reviewers. Any product that may be evaluated in this article, or claim that may be made by its manufacturer, is not guaranteed or endorsed by the publisher.
